# Supplementary material for: Structure of the DOCK2−ELMO1 complex provides insights into regulation of the auto-inhibited state
Source: Nat Commun. 2020 Jul 10;11:3464. doi: 10.1038/s41467-020-17271-9 (PMC7351999; doi:10.1038/s41467-020-17271-9)
Supplement: Supplementary file 1 — Supplementary Information [file 41467_2020_17271_MOESM1_ESM.pdf]

Structure of the DOCK2−ELMO1 complex provides insights into the regulation of the auto-inhibited state

Chang et al.

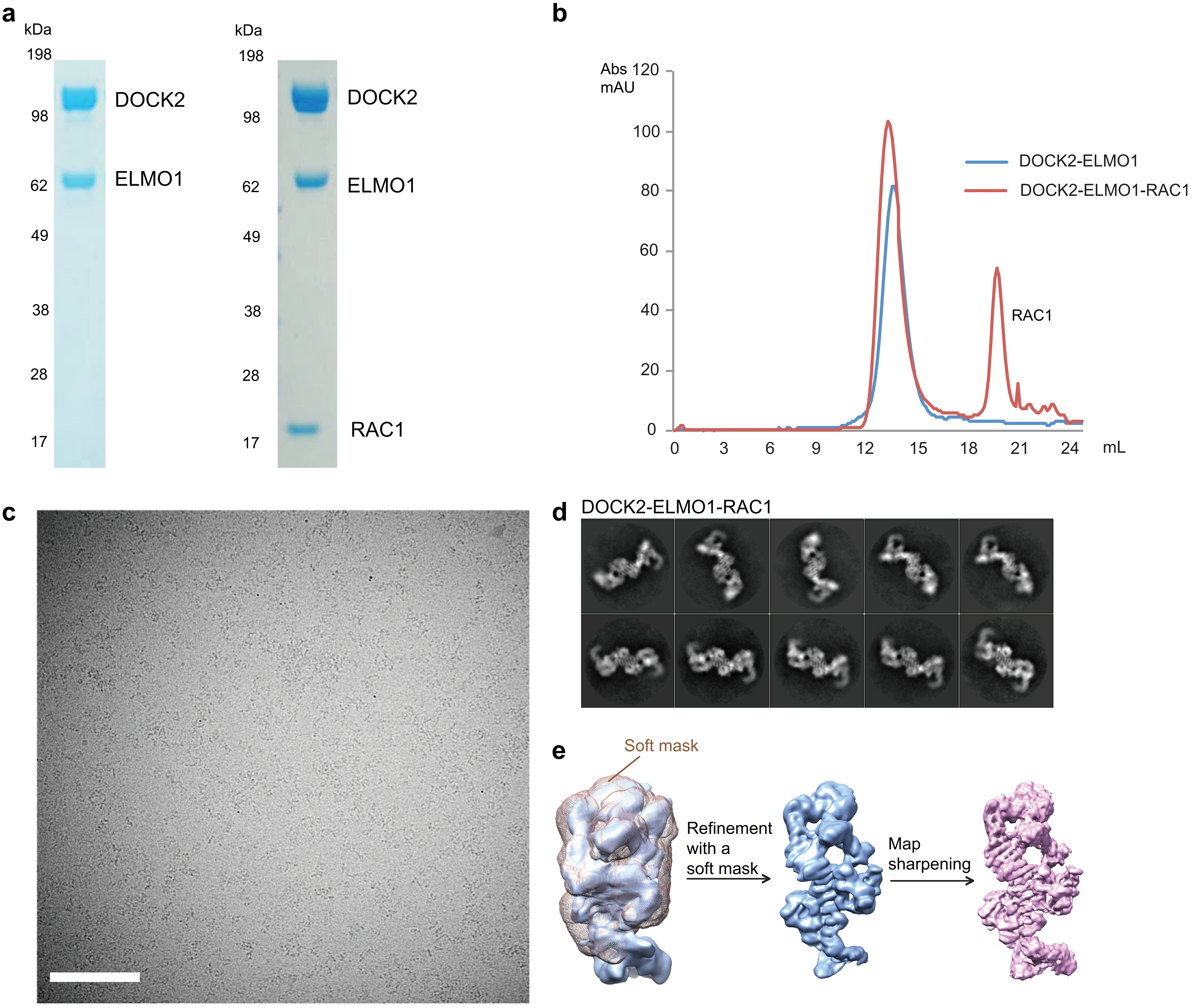

Supplementary Figure 1 | Sample preparation and cryo-EM of DOCK2−ELMO1 complexes. a Coomassie blue stained SDS-PAGE of the human DOCK2−ELMO1 **(left)** and DOCK2−ELMO1−RAC1 **(right)** complexes. The samples were not cross-linked. **b** Purification of DOCK2−ELMO1 (blue) and DOCK2−ELMO1−RAC1 (red) complexes by size-exclusion chromatography using Superose 6 Increase 10/300 GL. The experiments in (**a**) and (**b**) were performed independently three times with similar results. **c** A typical cryo-EM micrograph of the DOCK2−ELMO1−RAC1 complex, representative of 1914 micrographs. Scale bar = 100 nm. **d** Galleries of 2D class averages of DOCK2−ELMO1−RAC1, representative of 100 2D classes. **e** 3D refinement focussed on a protomer with a mask generates an improved map, allowing observation of α-helices. Source data are provided as a Source Data file.

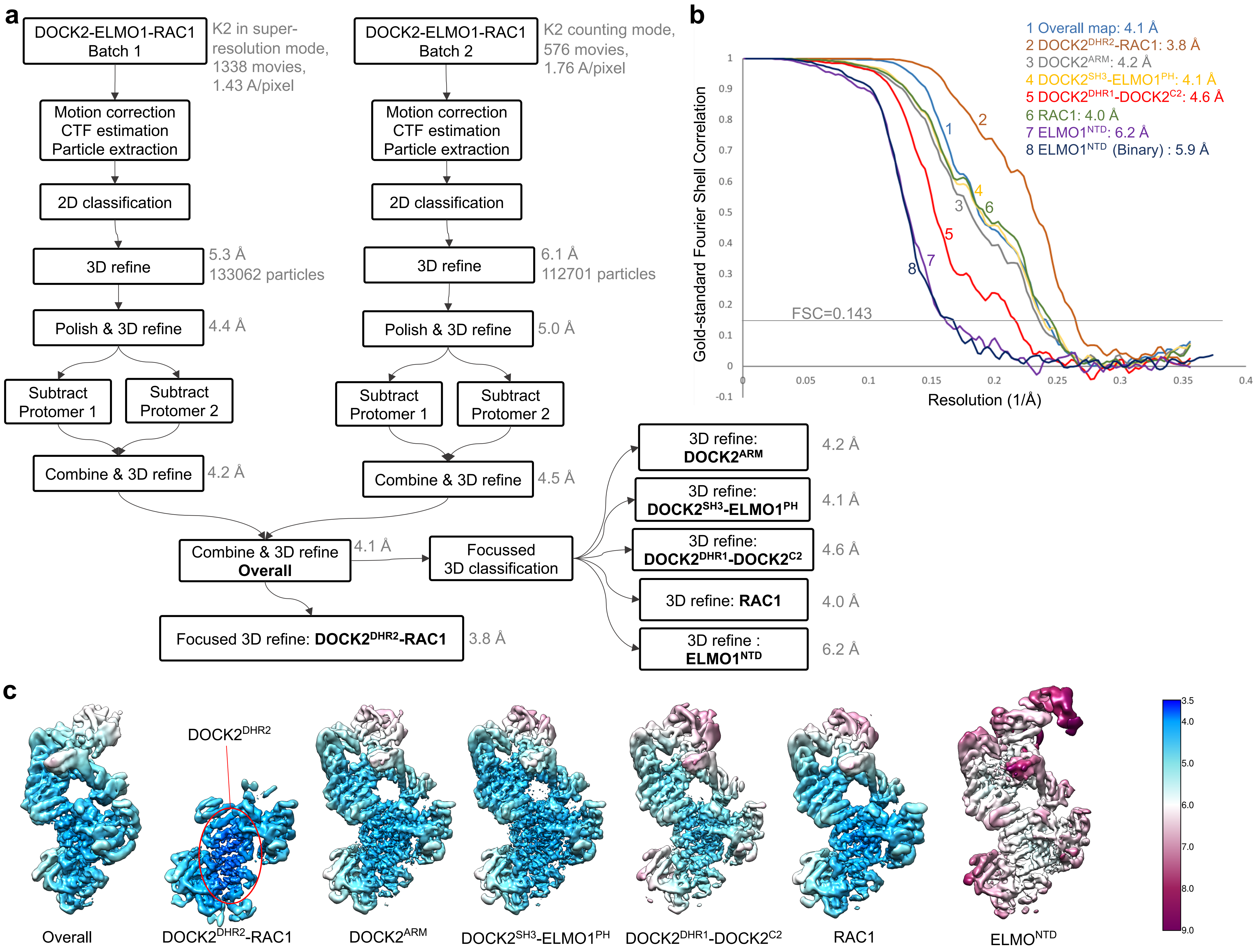

Supplementary Figure 2 | Cryo-EM data analysis. a Data processing flowchart for the DOCK2−ELMO1−RAC1 complex. See Methods for details. **b** Gold-standard FSC curves of cryo-EM maps for DOCK2−ELMO1 and DOCK2−ELMO1−RAC1 complexes. **c** Local resolutions of DOCK2−ELMO1−RAC1 maps were estimated using RELION [1-5]. Images were generated in Chimera [6].

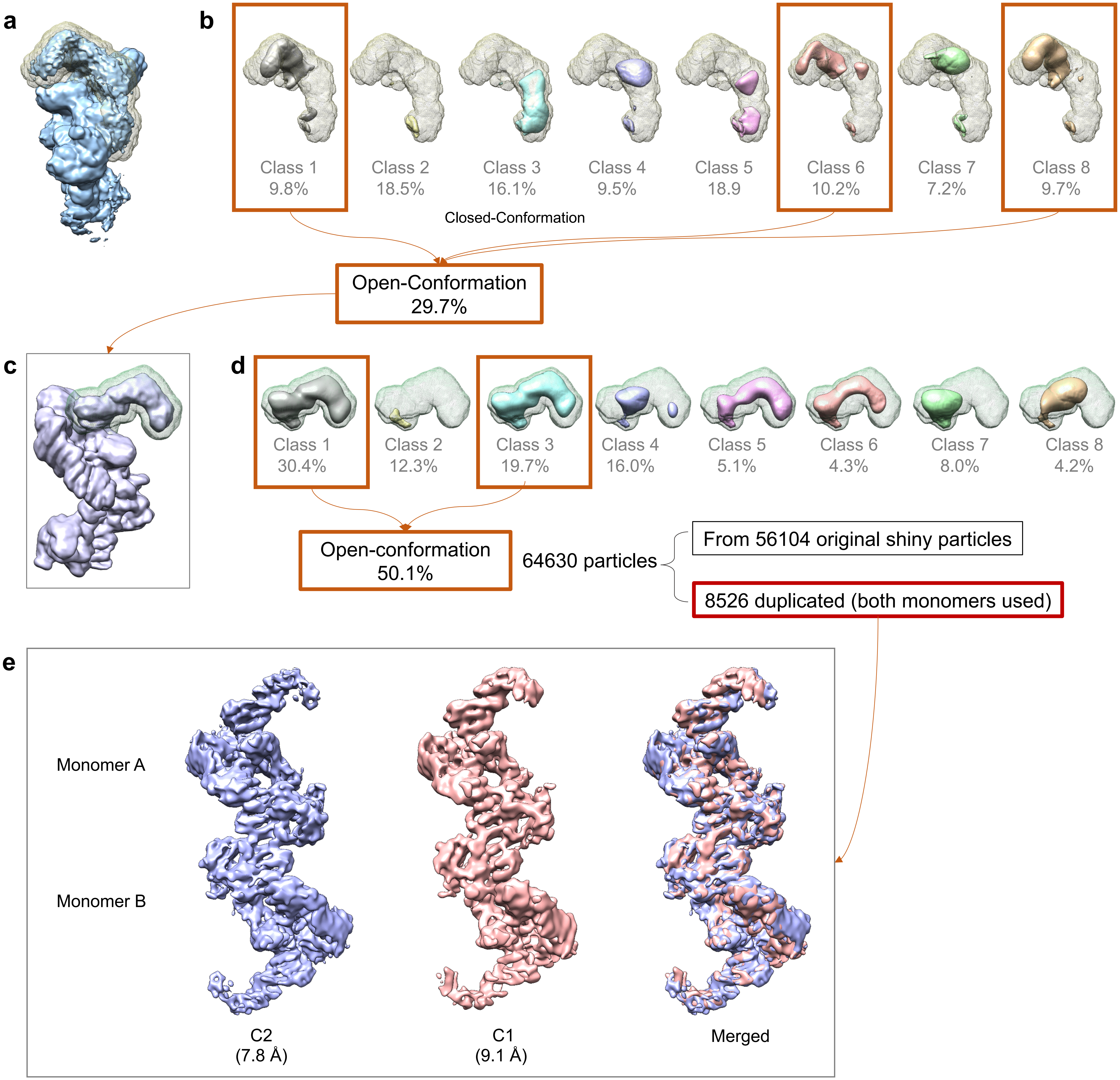

Supplementary Figure 3 | 3D classification of ELMO1^{NTD} in DOCK2−ELMO1−RAC1. a Overall reconstruction of the DOCK2−ELMO1−RAC1 protomer has weak densities for the ELMO1^{NTD}. A soft mask for the ELMO1^{NTD} is also shown in grey**. b** Focussed classification of ELMO1^{NTD} in the DOCK2−ELMO1−RAC1 complex, resulting in ~30% of particles in the open-conformation. **c** 3D reconstruction of selected particles with clear density for the ELMO1^{NTD}. **d** Second round of focussed classification for ELMO1^{NTD}. The first round made use of a bigger mask including ELMO1^{NTD} in both open- and closed- conformations, while the second round was focussed only for the open-conformation. **e** Particles with both ordered ELMO1^{NTD} domains were selected for a reconstruction of the whole DOCK2−ELMO1−RAC1 without imposed symmetry (C1) or with 2-fold symmetry (C2).

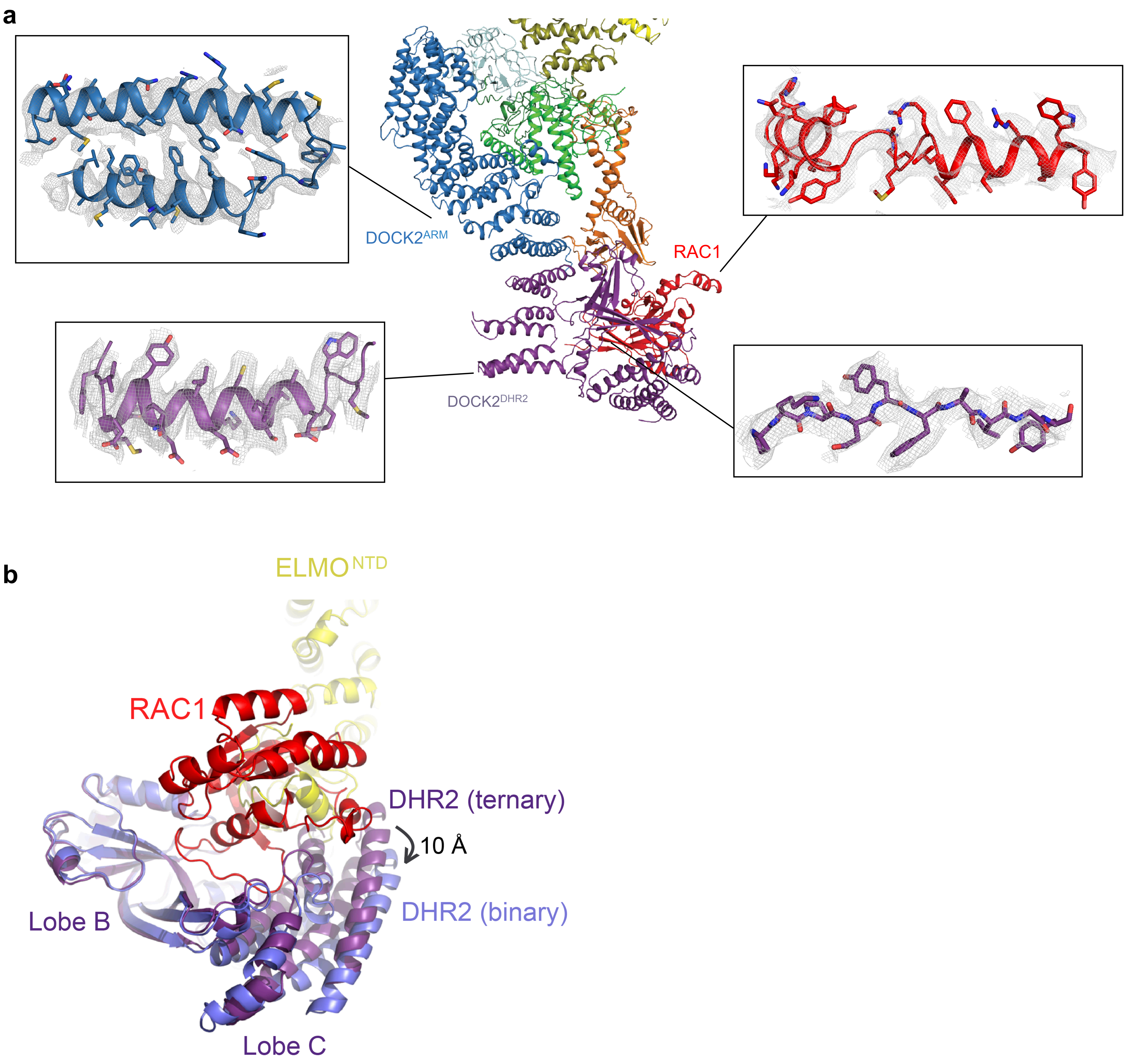

Supplementary Figure 4 | Typical densities and model building of DOCK2−ELMO1−RAC1 complex. a Representative density maps of the DOCK2^{DHR2} domain, RAC1, and DOCK2^{ARM} are shown. An α-helix, a β-strand a loop are presented. **b** Conformational change in lobe C of the DOCK2^{DHR2} domain between the binary and ternary complexes.

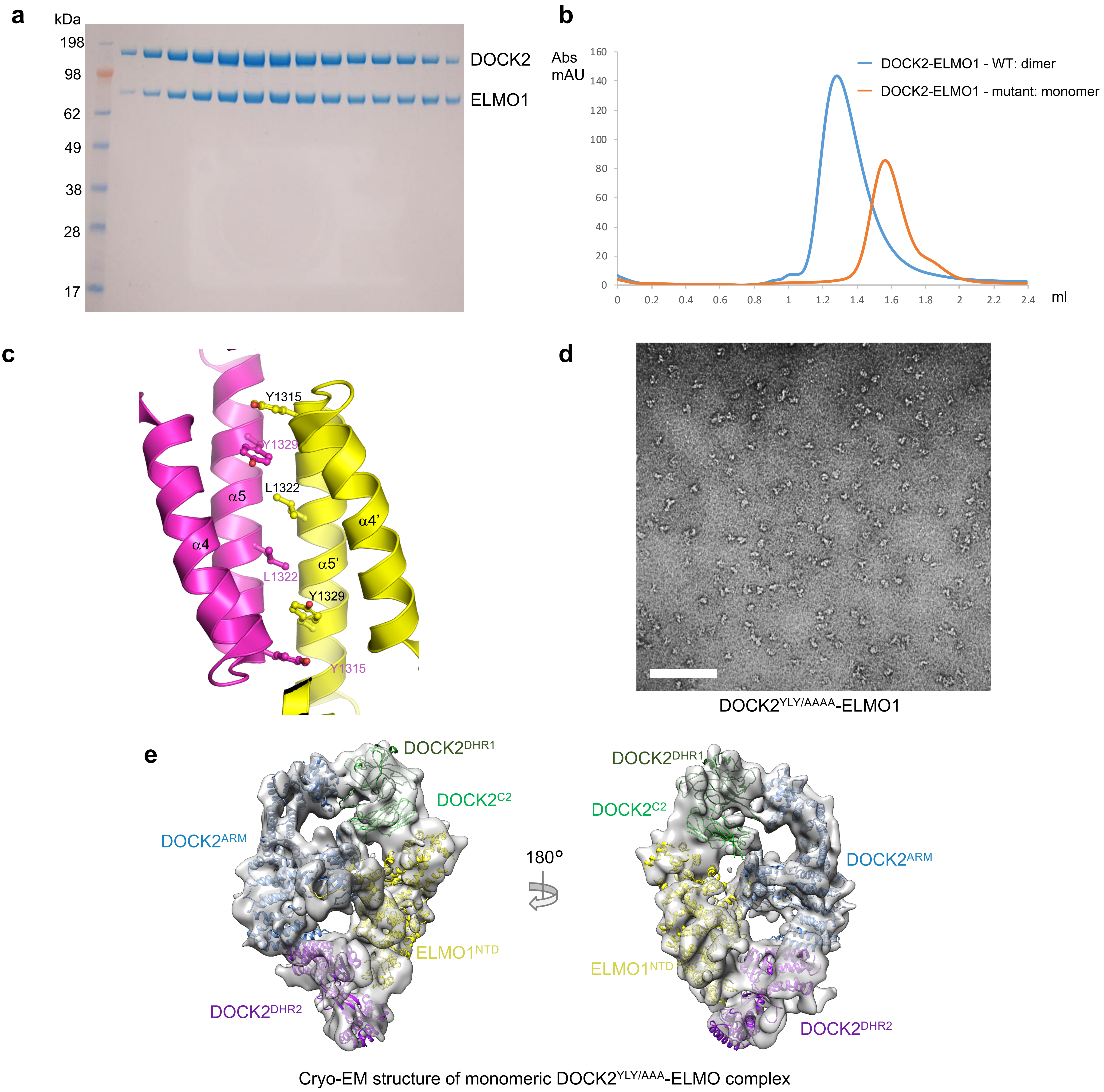

Supplementary Figure 5 | EM analysis of the monomeric DOCK2$^{YLY/AAA}$–ELMO1 complex. **a** Coomassie blue stained SDS-PAGE of the DOCK2$^{YLY/AAA}$–ELMO1 complex. The sample was not cross-linked. **b** SEC for the DOCK2–ELMO1 complex (wild type: dimer, light blue) and DOCK2–ELMO1 dimerization mutant (monomer, orange). The experiments in (**a**) and (**b**) were performed independently three times with similar results. **c** View of the DOCK2^{DHR2} dimerization interface showing dimer-interface mutants Y1315A/L1322A/Y1329A. Based on PDB 2YIN [46]. **d** A typical negative stain EM image of the DOCK2 $^{YLY/AAA}$ –ELMO1 complex, representative of 100 micrographs. Particles are smaller than the wild-type complex, indicating that they are not dimerized. Scale bar = 100 nm. **e** Cryo-EM map of the monomeric DOCK2 $^{YLY/AAA}$ –ELMO1 complex with DOCK2–ELMO1 model fitted. Source data are provided as a Source Data file.

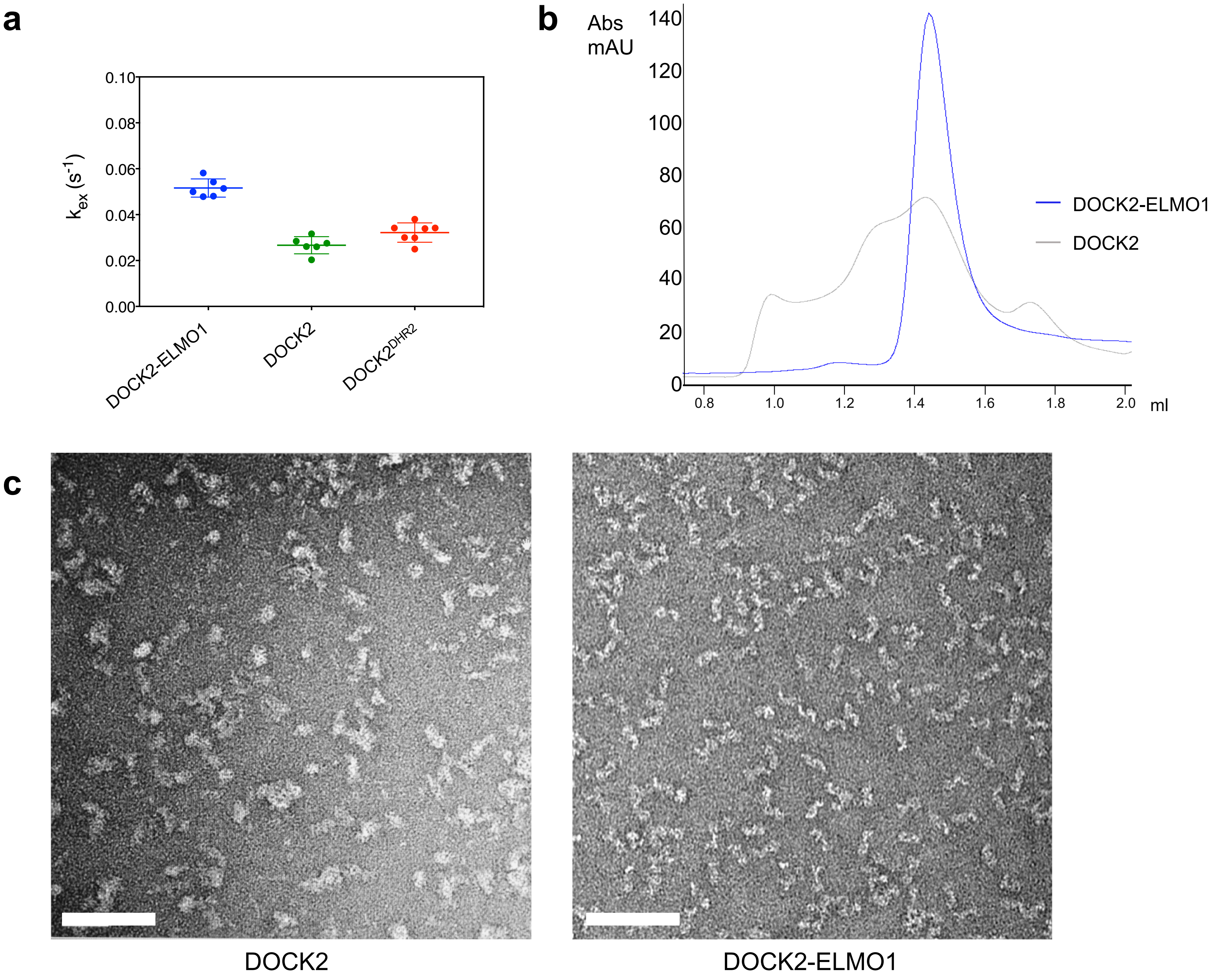

Supplementary Figure 6 | DOCK2 alone is unstable. a DOCK2 alone (green) showed reduced GEF activity compared to $DOCK2^{DHR2}$ (red). In contrast, the DOCK2−ELMO1 complex (blue) has twice the activity of DOCK alone. Data are presented as mean values with error bars of +/- one standard deviation. The experiment was performed seven times. k_{ex} is the rate constant for exchange of mant-GTP for GDP from a fit of the data to a single exponential rate equation to the entire exchange reaction. **b** Purification of DOCK2−ELMO1 (blue line) and DOCK2 alone (grey line) complexes by size-exclusion chromatography using Superose 6 Increase 3.2/300 GL. DOCK2 alone showed extended peaks. The experiment was performed independently three times with similar results. **c** Typical negative stain EM images of DOCK2 alone and the DOCK2−ELMO1 complex, representative of 40 and 205 micrographs, respectively. Particles of DOCK2 sample are heterogeneous in size. Large particles in the image are indicative of small aggregations. Scale bar = 100 nm. Source data are provided as a Source Data file.

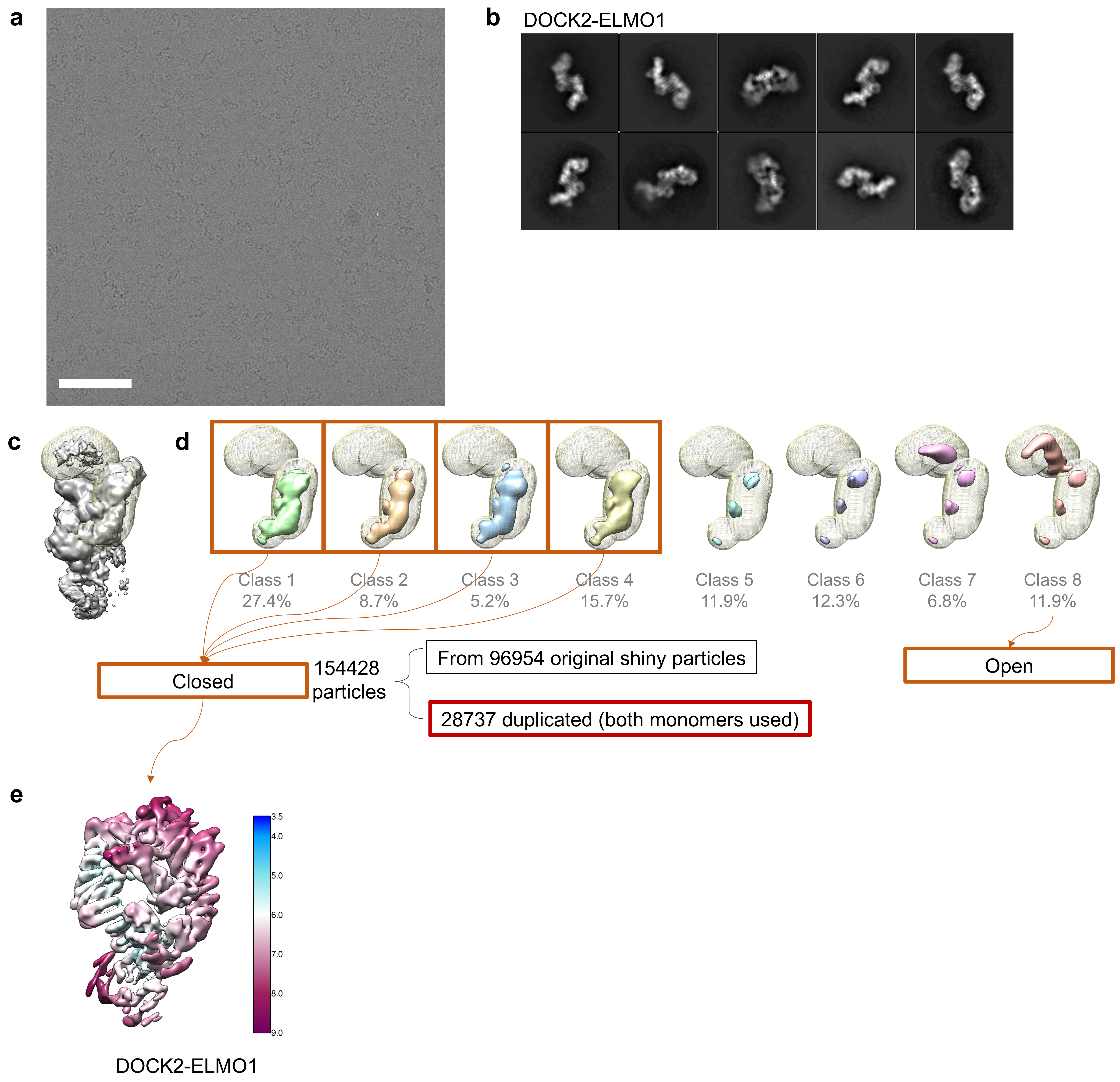

Supplementary Figure 7 | 3D reconstruction of DOCK2−ELMO1. **a** A typical cryo-EM micrograph of the DOCK2−ELMO1 complex, representative of 4591 micrographs. Scale bar = 100 nm. **b** Gallery of 2D class averages of DOCK2−ELMO1, representative of 100 2D classes. **c** Overall reconstruction of the DOCK2−ELMO1 protomer has weak densities for the ELMO1^{NTD}. A soft mask for the ELMO1^{NTD} is also shown**. d** Focussed classification of ELMO1^{NTD} in the DOCK2−ELMO1 complex, resulting in ~57% of particles in the closed-conformation and in ~12% of particles in the open-conformation. **e** Local resolutions of the DOCK2−ELMO1 map were estimated using RELION [1-5]. Images were generated in Chimera [6].

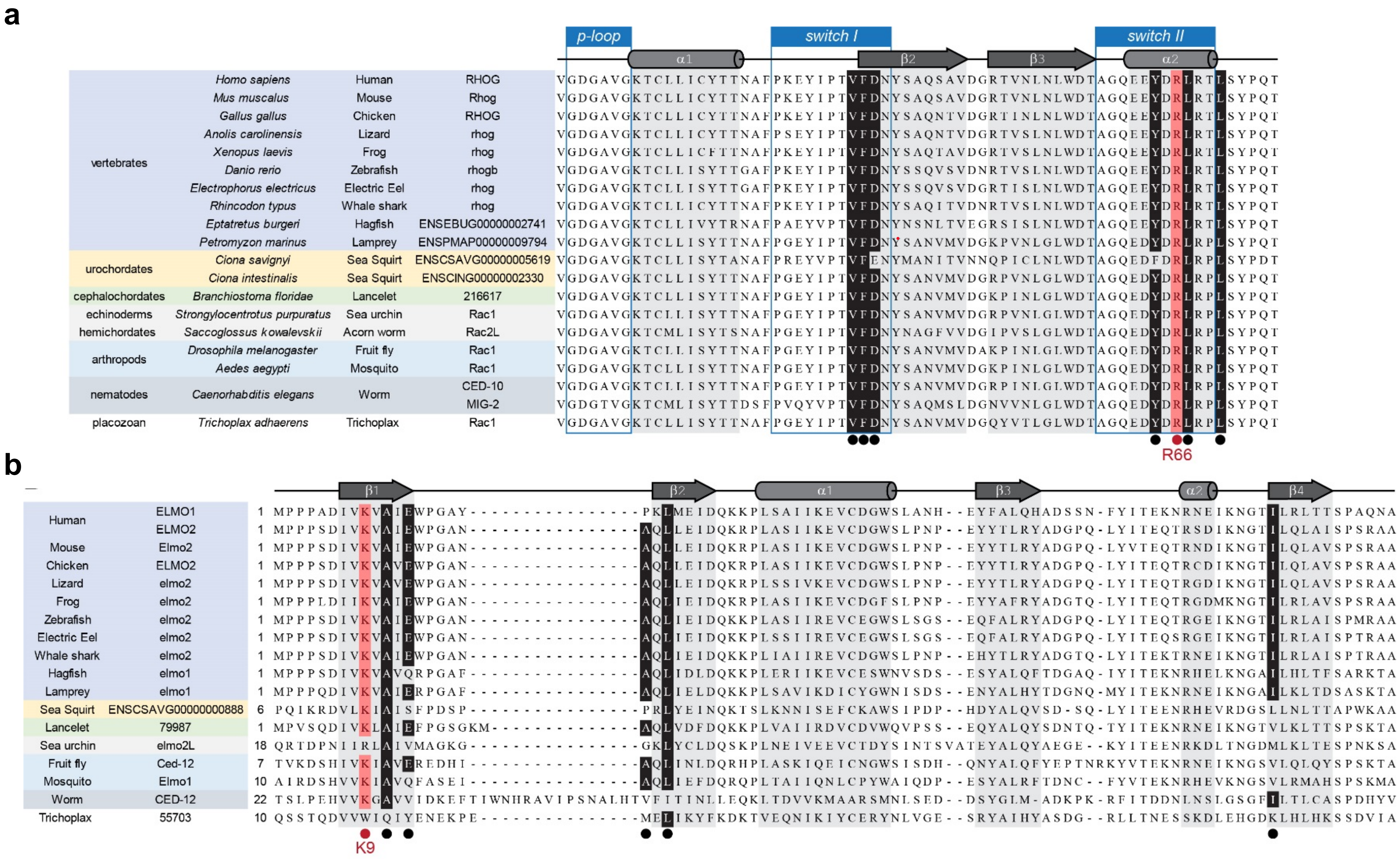

Supplementary Figure 8 | Sequence alignments demonstrating evolutionary conservation of RHOG and ELMO. RHOG (**a**) or ELMO2 (**b**) orthologs are aligned based on amino acid sequence. Location of secondary structures as derived from the RHOG−ELMO2^{RBD} structure are indicated at the top. The P-loop, switch 1 and switch 2 regions of RHOG are labelled over the secondary structures and outlined in blue boxes. Key residues at the binding interface are indicated with black points and shading. The important Lys9 residue of ELMO2 and Arg66 residue of RHOG are highlighted red.

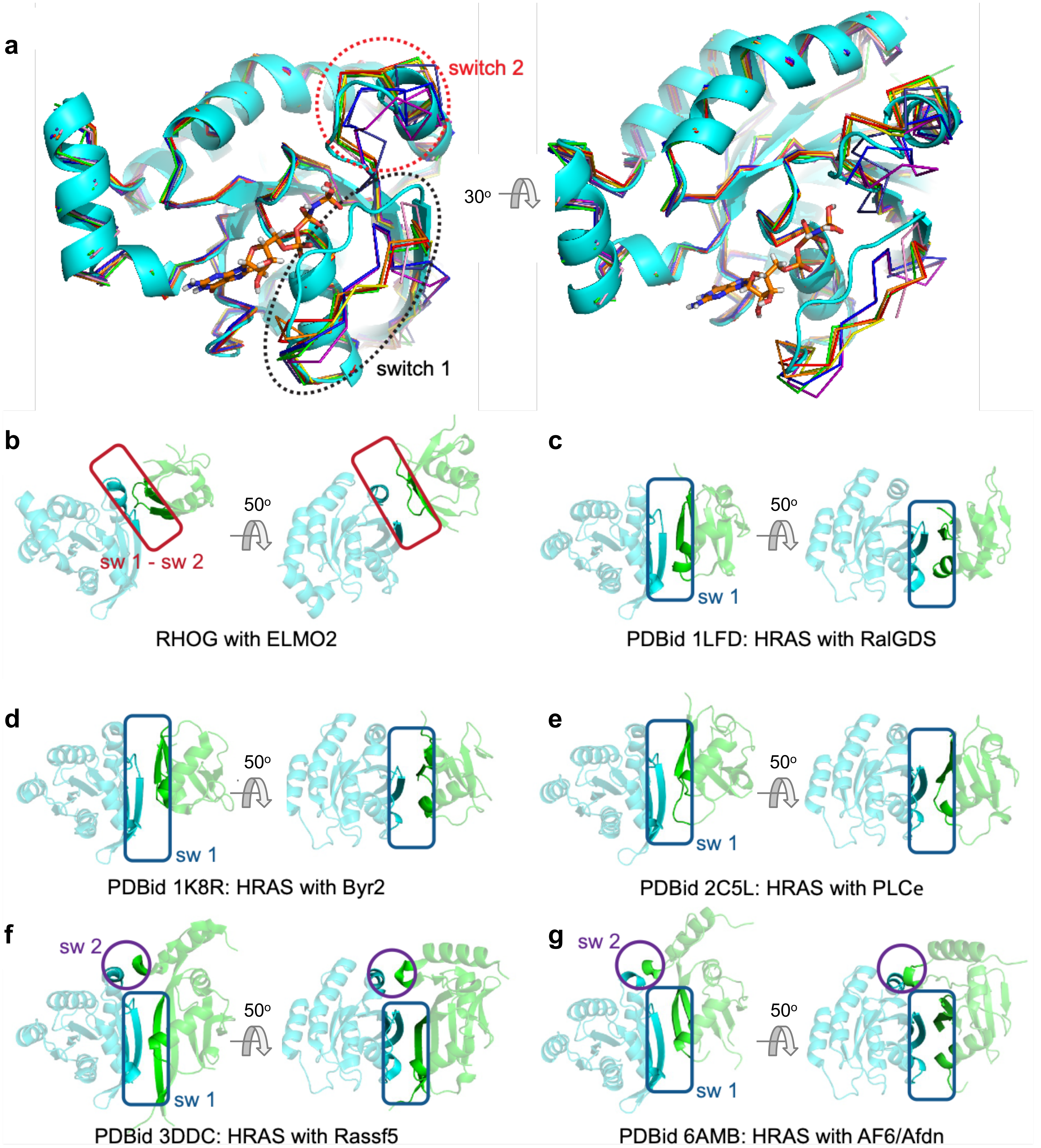

Supplementary Figure 9 | Potential impact of a RHOG nucleotide switch and comparison of the RHOG−ELMO2 interface with available HRAS−RBD complexes. **a** Structure alignment of GMPPNP-bound RHOG with other RHO family GTPases in the GDP-bound state. RHOG (cyan) is shown in cartoon and other RHO GTPases in ribbons form. These are RAC1 (PDB: 5N60, red), RAC2 (PDB: 2W2T, orange), RAC3 (PDB: 2C2H, yellow), CDC42 (PDB: 1AN0, green), RHOA (PDB: 1FTN, blue), RHOB (PDB: 2FV8, dark blue), RHOC (PDB: 2GCN, purple), and RHOU (PDB: 2Q3H, pink). The switch 1 and switch 2 regions are outlined in dotted lines. **b-g** Structure comparison of the interface between RHOG and the ELMO2 RBD versus HRAS bound to various ubiquitin-fold RBDs. All GTPases and RBDs are coloured cyan and green, respectively. Structures are depicted as ribbons and were aligned in 3D space based on position of the GTPase Intermolecular β-strand interactions between the RAS switch 1 region and RBDs are outlined in blue boxes, and non-canonical hydrophobic interactions with RAS switch 2 are circled in purple. The unique RHOG−ELMO2 interface which bridges switch 2 and 2 (**b**) is boxed in red; (**c**) is HRAS with RalGDS; (**d**) HRAS with Byr2; (**e**) HRAS with PLCε; (**f**) HRAS with Rassf5/Nore1; and (**g**) HRAS with AF6/Afdn.

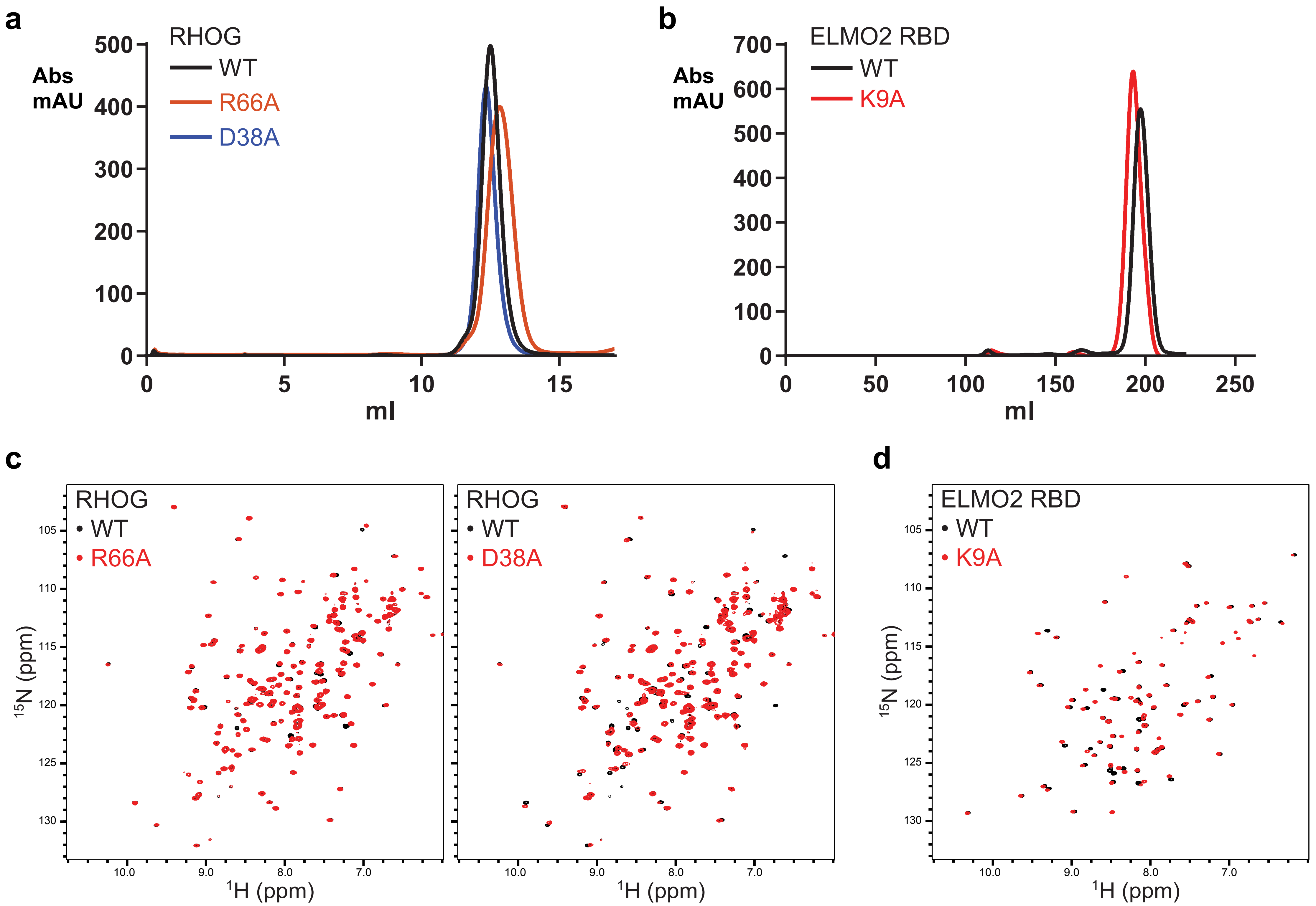

Supplementary Figure 10 | Folding and stability of RHOG GTPase and ELMO2^{RBD} wild-type and mutant proteins. a Overlay gel filtration (Superdex 75, 10/300 GL) chromatogram profiles of R66A (red) and D38A (blue) mutants of the RHOG GTPase with the wild-type protein (black). All proteins elute as monomers. **b** Overlay gel filtration (Superdex 75, High load 20/600) chromatogram profiles of wild-type (black) and the K9A (red) mutant of ELMO2^{RBD}. Both elute as monomers. Experiments in (**a**) and (**b**) were performed independently three times with results. **c** Overlay of ^{1}H-^{15}N HSQC spectra of ^{15}N-labelled, GMPPNP-loaded RHOG proteins (100 µM). Chemical shifts of the R66A (left, red) and D38A (right, red) mutant proteins overlay closely with wild-type RHOG (black), confirming they are properly folded in solution. **d** Overlay of ^{1}H-^{15}N HSQC spectra of ^{15}N-ELMO2^{RBD} (100 µM). Chemical shifts from the K9A mutant protein (red) coincide well with the wild-type RBD (black), verifying the mutant is properly folded in solution. Experiments in (**c**) and (**d**) were performed once.

Supplementary Table 1 | Cryo-EM data collection, refinement and validation statistics

	DOCK2-ELMO1-RAC1 – open conformation (EMDB-10498) (PDB 6TGC)	DOCK2-ELMO1 – closed conformation (EMDB-10497) (PDB 6TGB)
**Data collection and processing**		
Magnification	81,000	105,000
Voltage (kV)	300	300
Electron exposure (e–/Å^2)	40	40
Defocus range (μm)	1.5-3.2	1.5-3.2
Pixel size (Å)	1.43/1.76	1.76
Symmetry imposed	C1	C1
Initial particle images (no.)	245,763 dimers (491,526 protomers)	512,341 dimers (1,024,682 protomers)
Final particle images (no.)	417,684	22,311
Map resolution (Å)	4.13	5.99
FSC threshold	0.143	0.143
Map resolution range (Å)	7.80-3.90	9.25-5.00
**Refinement**		
Initial model used (PDB code)	3LN4C, 3A98, 2YIN, 6IDX	3LN4C, 3A98, 2YIN, 6IDX
Model resolution (Å)	4.1	5.5
FSC threshold	0.143	0.143
Model resolution range (Å)	50-3.9	50-5.0
Map sharpening *B* factor (Å^2)	-180	-300
Model composition		
Non-hydrogen atoms	32,728	18,161
Protein residues	5,038	2,342
Ligands	0	0
B factors (Å^2)		
Protein	X	X
Ligand	-	-
R.m.s. deviations		
Bond lengths (Å)	0.01	0.02
Bond angles (°)	1.05	1.59
Validation		
MolProbity score	-	-
Clashscore	95	95.03
Poor rotamers (%)	15	4.02
Ramachandran plot		
Favoured (%)	92.41	86.33
Allowed (%)	7.59	13.51
Disallowed (%)	0	0.16

Supplementary Table 2 | Cryo-EM focussed refinement parameters of domains of DOCK2-ELMO1 complexes

Reconstruction	DOCK2-ELMO1-RAC1								DOCK2-ELMO1		
Focussed region/domain	Overall	DOCK2^{DHR2}-RAC1	DOCK2^{ARM}	DOCK2^{SH3}-ELMO1^{PH}	DOCK2^{DHR1}-DOCK2^{C2}	RAC1	ELMO^{NTD}	Whole	Whole State 1	Whole State 2	ELMO1^{NTD}
Software	RELION 2.1								RELION 2.1		
Final particles	417684 Protomers	394718 Protomers	462039 Protomers	421810 Protomers	152820 Protomers	385220 Protomers	64630 Protomers	8526 Dimers	22311 Dimers	6426 Dimers	154428 Protomers
Accuracy of rotation alignment (degrees)	1.747	2.800	1.875	1.716	2.031	1.916	2.221	2.933	2.798	3.141	1.937
Accuracy of translation alignment (pixel)	0.933	1.198	1.008	0.927	1.014	0.956	1.159	1.639	2.001	2.344	1.336
Resolution	4.13	3.79	4.23	4.15	4.60	4.06	6.25	7.80	9.25	12.34	5.99
B-factor for sharpening	-180	-300	-160	-167	-160	-200	-200	-330	-1203	-1356	-255

Supplementary Table 3 | DOCK2-ELMO1-RAC1 domains

Subunit	Domain/segment	Residues	Coordinates	Reference
Human DOCK2 (1,830 amino acids)				
	SH3-helical domains	1-167	PDB 3A98 (DOCK2)	7
	α-helix	175-207	*De novo*	-
	C2 domain	219-421	ROSETTA model	-
	DHR1 domain	423-609	2	8
	ARM repeats	626-1194	*De novo*	-
	DHR2 domain	1196-1620	PDB 2YIN (DOCK2)	9
	PxxP/basic region	1621-1830	Disordered	-
Human ELMO1 (727 amino acids)				
	RBD-EID-ELMO	5-522	PDB 6IDX (ELMO2)	10
	Hinge helix	523-529	*De novo*	-
	PH domain	530-727	PDB 3A98	7
Human RAC1 (192 amino acids)				

Supplementary Table 4 | Crystallographic data collection and refinement statistics for ELMO2^{RBD}-RHOG

	ELMO2^{RBD}–RHOG Complex (PDB 6UKA)
Data collection	
Space group	C2
Cell dimensions	
a, b, c (Å)	100.3, 40.1, 73.9
α, β, γ (°)	90, 120, 90
Resolution (Å)	27-2.40 (2.44-2.40)
R_{merge}	0.105 (0.25)
$I / \sigma I$	9.77 (3.95)
Completeness (%)	92.7 (85.9)
Redundancy	3.0 (2.5)
Refinement	
Resolution (Å)	27.0-2.40
No. reflections	9436
R_{work} / R_{free}	18.56/23.66
No. atoms	
Protein	1938
Ligand/ion	49
Water	126
B-factors	
Protein	21.6
Ligand/ion	14.1
Water	22.9
R.m.s. deviations	
Bond lengths (Å)	0.003
Bond angles (°)	0.64

Values in parentheses are for highest-resolution shell.

Supplementary Table 5 | Primers used in this study

Oligonucleotide name	Oligo ref.	Oligonucleotide sequence	Purpose
hELMO1 attB Forward	1	5’ GGGGACAAGTTTGTACAAAAAAGCA GGCTTAATGCCACCACCTTCGGAC 3’	Gateway Cloning
hELMO1 attB Reverse	2	5’ GGGGACCACTTTGTACAAGAAAGCTGGG TTtcaGTTACAGTCATAGACAAAGTCATAG TTGCTAGGT 3’	Gateway Cloning
hELMO1 delta NTD attB Forward	3	5’ GGGGACAAGTTTGTACAAAAAAGCAGG CTTAATGTCTCGCCCGATTTTGGAAC 3’	Gateway Cloning
hELMO1 delta NTD attB Reverse	4	5’ GGGGACCACTTTGTACAAGAAAGCTGG GTTtcaGTTACAGTCATAGACAAAGTCATAGTTGC 3’	Gateway Cloning
Vector Forward	5	5’ GCTCTCAGTGGCAGGCATCCCTGGGT TGGATG 3’	HiFi Gibson Assembly – DOCK2
Vector Reverse	6	5’ AAGGTTCCTGAGAGAAGCTGGGACC TCCACTC 3’	HiFi Gibson Assembly – DOCK2
Fragment 1 Forward	7	5’ ATCTGATGGAGTGGAGGTCCCAGCT TCTCTCA 3’	HiFi Gibson Assembly – DOCK2
Fragment 1 Reverse	8	5’ CGACCCGGACTATTTGACAAATCAA GTAAATT 3’	HiFi Gibson Assembly – DOCK2
Fragment 2 Forward	9	5’ AGGGATAAAATTTACTTGATTTGTC AAATAGTCCGG 3’	HiFi Gibson Assembly – DOCK2
Fragment 2 Reverse	10	5’ AGAACGGTGTTGAAATGCTGAAAT TTCCGGTC 3’	HiFi Gibson Assembly – DOCK2
Fragment 3 Forward	11	5’ TCATTGCAGACCGGAAATTTCAGC ATTTCAAC 3’	HiFi Gibson Assembly – DOCK2
Fragment 3 Reverse	12	5’ GGGATAAGTGTCATCTCTAATATA GGTCCTACC 3’	HiFi Gibson Assembly – DOCK2
Fragment 4 Forward	13	5’ CAGGCATGGTAGGACCTATATTAGA GATGACA 3’	HiFi Gibson Assembly – DOCK2
Fragment 4 Reverse	14	5’ GTTGGCCTCATCCAACCCAGGGAT GCCTGCCA 3’	HiFi Gibson Assembly – DOCK2
RHOG attB Forward	15	5’ GGGGACAAGTTTGTACAAAAAAGCAGGCTCCCTG GTGCCGCGCGGCAGCATGCAGAGCATCAAGTGCG 3’	Gateway Cloning
RHOG attB Reverse	16	5’ GGGGACCACTTTGTACAAGAAAGCTG GGTCTCACAAGAGGATGCAGGACC 3’	Gateway Cloning
RHOG D38A Top	17	5’ CCACCGTGTTCGCCAATTACAGCGC 3’	Mutagenesis

RHOG D38A Bottom	18	5’ GCGCTGTAATTGGCGAACACGGTGG 3’	Mutagenesis
RHOG R66A Top	19	5’ CAGGAGGAGTATGACGCCCTCCGTACACTCTC 3’	Mutagenesis
RHOG R66A Bottom	20	5’ GAGAGTGTACGGAGGGCGTCATACTCCTCCTG 3’	Mutagenesis
hELMO2 K9A Top	21	5’ CGTCTGACATTGTCGCAGTGGCCATCGAG 3’	Mutagenesis
hELMO2 K9A Bottom	22	5’ CTCGATGGCCACTGCGACAATGTCAGACG 3’	Mutagenesis
pos6R	23	5’ ATTTCCGUGTCGCCCTTATTC3’	DOCK2DHR2 avi tag pOPIN*
pos7F	24	5’ACGGAAAUGTTGAATACTCATACTCTTC3’	DOCK2DHR2 avi tag pOPIN*
DOCK2aviR	25	5’ ATGCCACUCGATTTTTTGAGCTTCAAAAATATCATTAAGACCCTG GTAGTACAGGTTTTCGG3’	DOCK2DHR2 avi tag pOPIN*
DOCK2aviF	26	5’ AGTGGCAUGAGGGTTCTGCTGGTGAATGCGGAGATATG ACAGATG3’	DOCK2DHR2 avi tag pOPIN*
Ppr(r1)	27	5’ ATATTTATAGGUTTTTTATTACAAAACTGTTAC3’	ELMO1ΔNTD
elmoNdeIF	28	5’ ACCTATAAATAUGTCTCGCCCGATTTTGGAACT3’	ELMO1ΔNTD
elmoaviR	29	5’ ATCTTCTGUGCTTCAAAAATATCATTAAGACCAGCACCACTG TTACAGTCATAG3’	ELMO1ΔNTD
elmoaviF	30	5’ ACAGAAGAUTGAATGGCATGAAGAGAATTTGTAT TTTCAAAGTTGGAG 3’	ELMO1Δ NTD
Dk2DDR	31	5’ ATAGCGTTCUGGCTGAGCAGCTCAGCGTCAAAGATCTCCATCT	DOCK2 Y1315A/L1322A/Y1329A
Dk2DDF	32	5’ AGAACGCTAUCCAGCAGGCAAAATTCGCTGAAA GCATCATGAAAATC	DOCK2 Y1315A/L1322A/Y1329A
Ubr	33	5’ AGCATGCGGUCTACGGGTACGTTTAAACCAGA	DOCK2 Y1315A/L1322A/Y1329A
1Fubase	34	5’ ACCGCATGCUTGGCTATGGCAGGGCTTGC	DOCK2 Y1315A/L1322A/Y1329A
Ppr(r1)	35	5’ ATATTTATAGGUTTTTTTATTACAAAACTGTTAC	DOCK2 cloning
Dk2flF	36	5’ ACCTATAAATAUGGGTAGCAGATATACCATGA	DOCK2 cloning
UCDMF	37	5’ ACAAGCTu GTCGAGA AGTACTAG	DOCK2 cloning
Ppr(r1)	38	5’ ATATTTATAGGu TTTTTTAT TACAAAACTG TTAC	DOCK2 cloning
Dk2flF	39	5’ ACCTATAAATAug GGTAGC AGATATACCA TGA	DOCK2 cloning

D2flR2	40	5’ ATGGCTGuCA TACATGCCAC AAA	DOCK2 cloning
D2flF2	41	5’ ACAGCCAu CTTAAACCAGATGG	DOCK2 cloning
Dk2flR	42	5’ AAGCTTGu ta GTGAt GGTGATGGTG ATGAT	DOCK2 cloning
TevelmoF	43	5’ AGTGGTGCu GAGAATTTGTATTTTCAAAG TTGGAGC	ELMO1 cloning
Ppr(r1)	44	5’ ATATTTATAGGu TTTTTTAT TACAAAACTG TTAC	ELMO1 cloning
elmoF	45	5’ AC CTATAAATaug CCaC CaCCTTCGGACATCGTGAAG	ELMO1 cloning
ElmoR	46	5’ AGCACCACuG TTACAGTCAT AGACAAAGTC ATAG	ELMO1 cloning

Supplementary References

1 Fernandez-Leiro, R. & Scheres, S. H. W. A pipeline approach to single-particle processing in RELION. *Acta crystallographica. Section D, Structural biology* **73**, 496-502, (2017).

2 Nakane, T., Kimanius, D., Lindahl, E. & Scheres, S. H. Characterisation of molecular motions in cryo-EM single-particle data by multi-body refinement in RELION. *eLife* **7**, doi:10.7554/eLife.36861 (2018).

3 Scheres, S. H. RELION: implementation of a Bayesian approach to cryo-EM structure determination. *J Struct Biol* **180**, 519-530, (2012).

4 Scheres, S. H. Semi-automated selection of cryo-EM particles in RELION-1.3. *Journal of structural biology* **189**, 114-122, (2015).

5 Bai, X. C., Rajendra, E., Yang, G., Shi, Y. & Scheres, S. H. Sampling the conformational space of the catalytic subunit of human gamma-secretase. *eLife* **4**, doi:10.7554/eLife.11182 (2015).

6 Yang, Z. *et al.* UCSF Chimera, MODELLER, and IMP: an integrated modeling system. *J Struct Biol* **179**, 269-278, (2012).

7 Hanawa-Suetsugu, K. *et al.* Structural basis for mutual relief of the Rac guanine nucleotide exchange factor DOCK2 and its partner ELMO1 from their autoinhibited forms. *Proceedings of the National Academy of Sciences of the United States of America* **109**, 3305-3310, (2012).

8 Premkumar, L. *et al.* Structural basis of membrane targeting by the Dock180 family of Rho family guanine exchange factors (Rho-GEFs). *The Journal of biological chemistry* **285**, 13211-13222, (2010).

9 Kulkarni, K., Yang, J., Zhang, Z. & Barford, D. Multiple Factors Confer Specific Cdc42 and Rac Protein Activation by Dedicator of Cytokinesis (DOCK) Nucleotide Exchange Factors. *The Journal of biological chemistry* **286**, 25341-25351, 2011).

10 Weng, Z. *et al.* Structure of BAI1/ELMO2 complex reveals an action mechanism of adhesion GPCRs via ELMO family scaffolds. *Nature communications* **10**, 51, doi:10.1038/s41467-018-07938-9 (2019).
