# Supplementary material for: Structure of the DOCK2−ELMO1 complex provides insights into regulation of the auto-inhibited state
Source: Nat Commun. 2020 Jul 10;11:3464. doi: 10.1038/s41467-020-17271-9 (PMC7351999; doi:10.1038/s41467-020-17271-9)
Supplement: Supplementary file 3 — Description of Additional Supplementary Files [file 41467_2020_17271_MOESM3_ESM.pdf]

Description of Additional Supplementary Files

File Name: Supplementary Movie 1
Description: Conformational change of ELMO1. Movie showing morphing between the open- and closed- (auto-inhibited) conformations of the DOCK2–ELMO1 complex. EM density for the open-conformation is in light blue and yellow for the closed-conformation.

File Name: Supplementary Movie 2
Description: ELMO-NTD is required for cell motility. Comparison of phase contrast bright-field live-cell imaging videos of HeLa cells expressing the indicated plasmids imaged using time-lapse microscopy for 6 h.
