# Supplementary material for: Structure of the DOCK2−ELMO1 complex provides insights into regulation of the auto-inhibited state
Source: Nat Commun. 2020 Jul 10;11:3464. doi: 10.1038/s41467-020-17271-9 (PMC7351999; doi:10.1038/s41467-020-17271-9)
Supplement: Supplementary file 6 — Reporting Summary [file 41467_2020_17271_MOESM6_ESM.pdf]

nature research

Corresponding author(s): David Barford

Last updated by author(s): Jun 5, 2020

Reporting Summary

Nature Research wishes to improve the reproducibility of the work that we publish. This form provides structure for consistency and transparency in reporting. For further information on Nature Research policies, see Authors & Referees and the Editorial Policy Checklist .

Statistics

For all statistical analyses, confirm that the following items are present in the figure legend, table legend, main text, or Methods section.

n/a	Confirmed	
[ ]	[x]	The exact sample size (n) for each experimental group/condition, given as a discrete number and unit of measurement
[x]	[ ]	A statement on whether measurements were taken from distinct samples or whether the same sample was measured repeatedly
[ ]	[x]	The statistical test(s) used AND whether they are one- or two-sided<br>*Only common tests should be described solely by name; describe more complex techniques in the Methods section.*
[x]	[ ]	A description of all covariates tested
[x]	[ ]	A description of any assumptions or corrections, such as tests of normality and adjustment for multiple comparisons
[ ]	[x]	A full description of the statistical parameters including central tendency (e.g. means) or other basic estimates (e.g. regression coefficient) AND variation (e.g. standard deviation) or associated estimates of uncertainty (e.g. confidence intervals)
[ ]	[x]	For null hypothesis testing, the test statistic (e.g. F, t, r) with confidence intervals, effect sizes, degrees of freedom and P value noted<br>*Give P values as exact values whenever suitable.*
[x]	[ ]	For Bayesian analysis, information on the choice of priors and Markov chain Monte Carlo settings
[x]	[ ]	For hierarchical and complex designs, identification of the appropriate level for tests and full reporting of outcomes
[x]	[ ]	Estimates of effect sizes (e.g. Cohen's d, Pearson's r), indicating how they were calculated

Our web collection on statistics for biologists contains articles on many of the points above.

Software and code

Policy information about availability of computer code

Data collection	Commercial software: EPU (ver 2.6) from Thermo Fisher Scientific was used for automated cryo-EM data collection. X-ray crystallographic data were collection using Proteum (ver 3) (Bruker).
Data analysis	Cryo-EM data were analysed using the software MotionCor2 (ver 2.1), GCTF (ver 0.5), EMAN2 (ver 2.31) and RELION (vers 1.4, 2.1 and 3.0). Model building and refinement were using COOT (ver 0.8.9.2) and Phenix (ver 1.18.2) and validated using MolProbility (ver 4.2). Visualization was performed with Pymol (ver 2.3.3) and Chimera (ver 1.14). X-ray crystallographic data were analysed using Phenix (ver 1.18.2).<br>Protein structure predictions were performed with the PHYRE2 and I-TASSER web tools<br>Isothermal titration calorimetry data were fit using the Origin (ver 7) software (OriginLab Corporation).<br>Densitometry analysis was performed using ImageJ software program (ver 1.52j).<br>GEF activity data were analysed using GraphPad PRISM (ver 8.2.1).

For manuscripts utilizing custom algorithms or software that are central to the research but not yet described in published literature, software must be made available to editors/reviewers. We strongly encourage code deposition in a community repository (e.g. GitHub). See the Nature Research guidelines for submitting code & software for further information.

Data

Policy information about availability of data

All manuscripts must include a data availability statement. This statement should provide the following information, where applicable:

- Accession codes, unique identifiers, or web links for publicly available datasets
- A list of figures that have associated raw data
- A description of any restrictions on data availability

EM maps are deposited in the Electron Microscopy Data Bank under accession codes: 10498 (DOCK2–ELMO1–RAC1 ternary complex, open conformation) [https://

www.ebi.ac.uk/pdbe/entry/emdb/EMD-10498], 10497 (DOCK2–ELMO1 binary complex, closed conformation) [https://www.ebi.ac.uk/pdbe/entry/emdb/]EMD-10497]. Protein coordinates are deposited in the Protein Data Bank under accession codes: 6TGC (DOCK2–ELMO1–RAC1 ternary complex, open conformation), 6TGB (DOCK2–ELMO1–RAC1 ternary complex, open conformation) and 6UKA (ELMO2RBD–RHOG). The source data underlying Figs. 6b, 7b-f, 8b-g and Supplementary Figs. 1a, 5a, 6a are provided as a Source Data file. Plasmids and cell lines that were generated for and used in this study are available upon request from the authors.

Field-specific reporting

Please select the one below that is the best fit for your research. If you are not sure, read the appropriate sections before making your selection.

☒ Life sciences ☐ Behavioural & social sciences ☐ Ecological, evolutionary & environmental sciences

For a reference copy of the document with all sections, see nature.com/documents/nr-reporting-summary-flat.pdf

Life sciences study design

All studies must disclose on these points even when the disclosure is negative.

Sample size	We collected 1,914 cryo-EM images for the DOCK2-ELMO1-RAC1 dataset and 4,591 cryo-EM images for the DOCK2-ELMO1 dataset. The total number of particles for the DOCK2-ELMO1-RAC1 data set was 417,684 and that for the DOCK2-ELMO1 data set was 154,428. Sample sizes were estimated on the basis of previous studies using similar methods that are widely publicized.<br><br>For the Boyden migration and invasion assay, 100,000 cells were seeded and allowed to migrate. For time-lapse cell imaging 1000 cells/well, in a 12 well plate were used. The number of cells was chosen based on previous publications in the field. The experimental data are the result of independent triplicates.
Data exclusions	No data were excluded from the analysis.
Replication	All attempts at replication were successful and reproducible. At least three independent biological repeats per experiment where representative data are shown. P-values are stated to show the statistical significance. Structure determination does not require replication.
Randomization	Samples were not allocated into groups. Cell cultures were randomly assigned to different groups and treatments.
Blinding	Blinding was not relevant to this study because there was no group allocation.

Reporting for specific materials, systems and methods

We require information from authors about some types of materials, experimental systems and methods used in many studies. Here, indicate whether each material, system or method listed is relevant to your study. If you are not sure if a list item applies to your research, read the appropriate section before selecting a response.

Materials & experimental systems

n/a	Involved in the study
☐	☒ Antibodies
☐	☒ Eukaryotic cell lines
☒	☐ Palaeontology
☒	☐ Animals and other organisms
☒	☐ Human research participants
☒	☐ Clinical data

Methods

n/a	Involved in the study
☒	☐ ChIP-seq
☒	☐ Flow cytometry
☒	☐ MRI-based neuroimaging

Antibodies

Antibodies used	Anti-Myc (Santa Cruz - SC40), anti-FLAG-M2 HRP (Sigma - A8592), anti-RAC1 (EMD MILLIPORE - 05389).
Validation	Antibodies used in this study were obtained from commercial suppliers.<br>Anti-Myc 9E10 (Santa Cruz - SC40) was validated against expressed Myc protein in HeLa cells by Santa Cruz and is widely used in the field.<br>Anti-FLAG-M2 (Sigma - A8592) was validated on recombinant FLAG peptide by Sigma and widely used in the field.<br>Anti-RAC1 (EMD MILLIPORE - 05389) was validated on rat brain microsomes by EMD.

Eukaryotic cell lines

Policy information about cell lines

Cell line source(s)	HEK 293T and HeLa cells were obtained from ATCC<br>High Five™ Cells (BTI-TN-5B1-4)], ThermoFisher Cat no. B85502.
Authentication	None of the cell lines were authenticated
Mycoplasma contamination	The cell lines were not routinely tested for mycoplasma contamination.
Commonly misidentified lines<br>(See ICLAC register)	None
